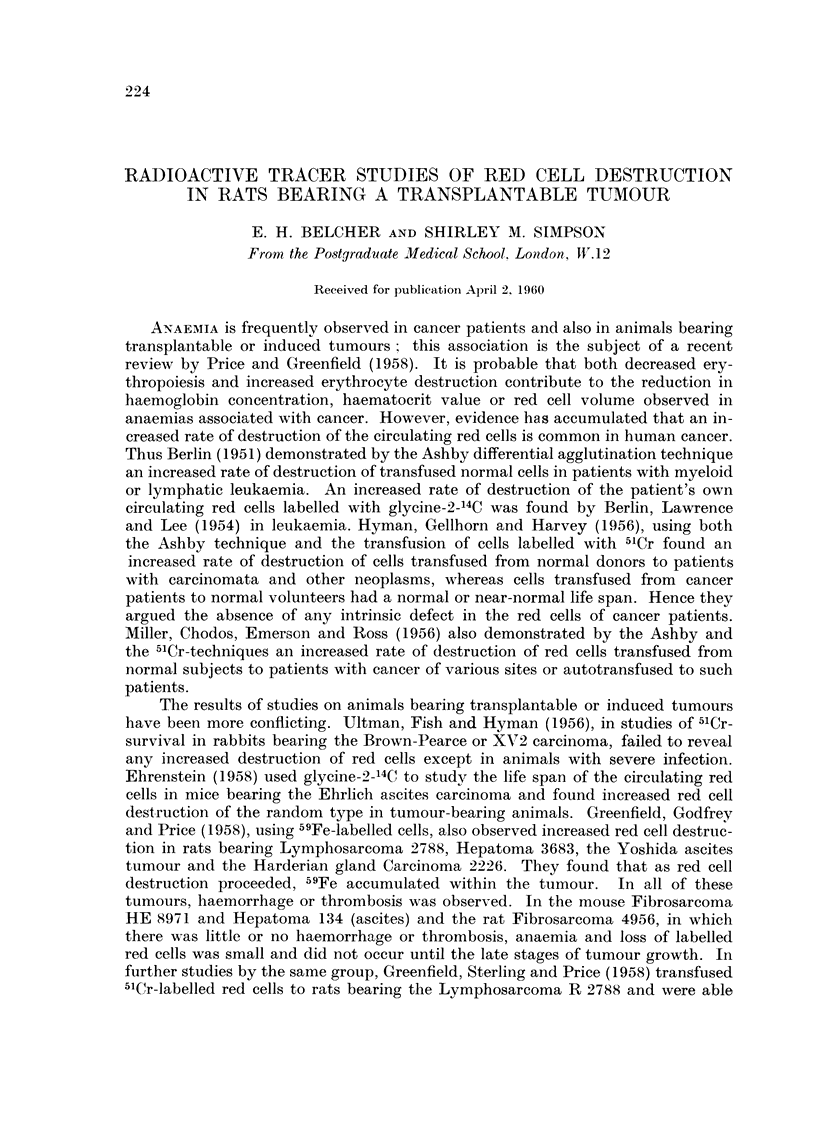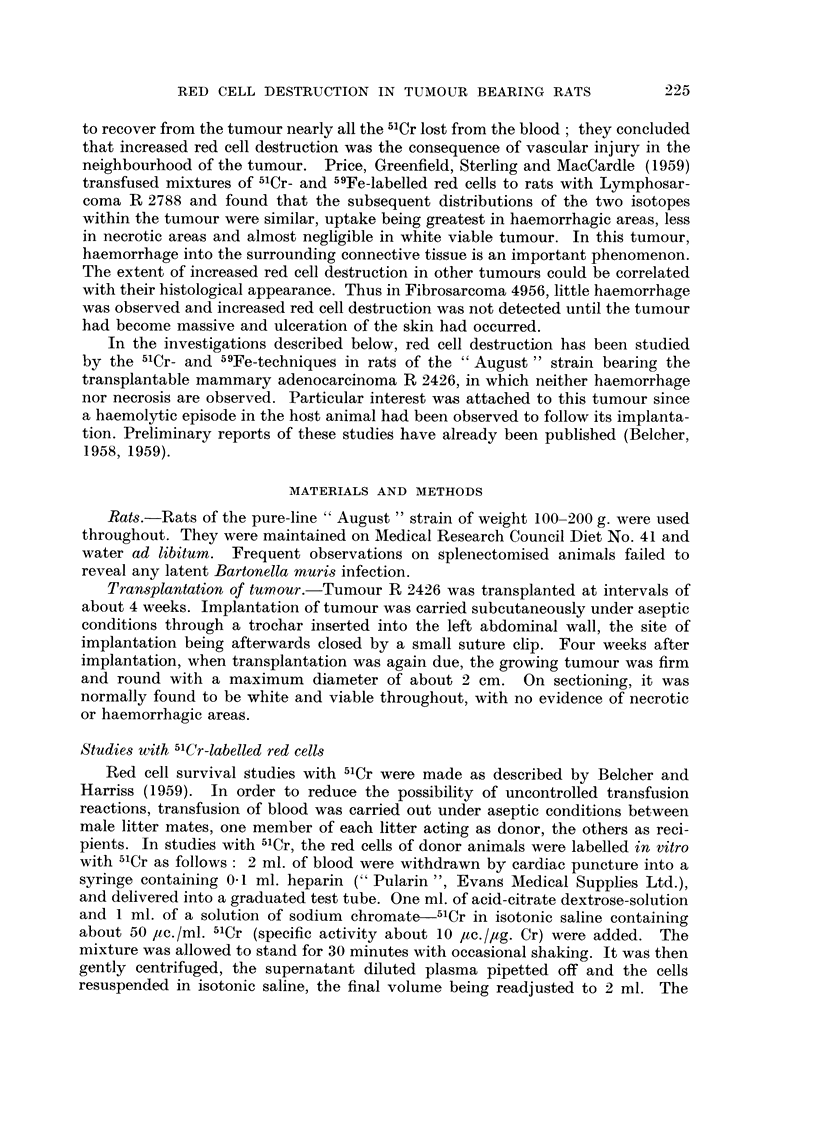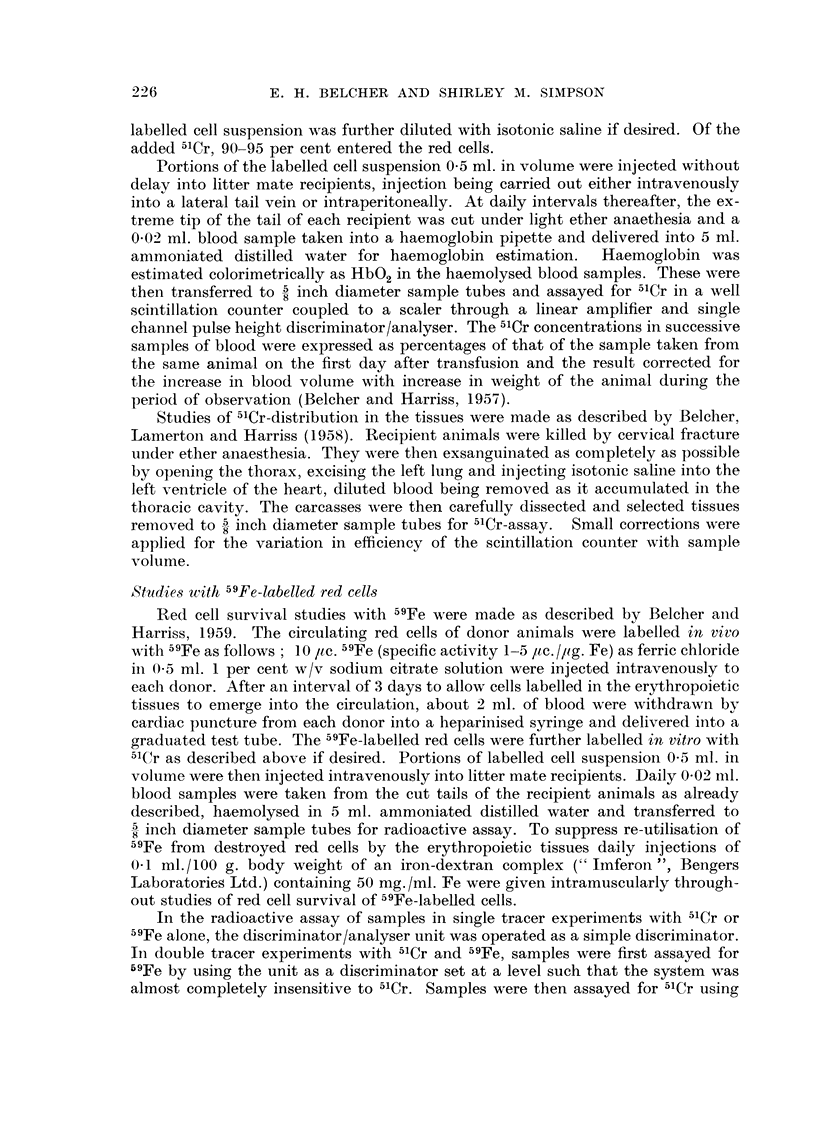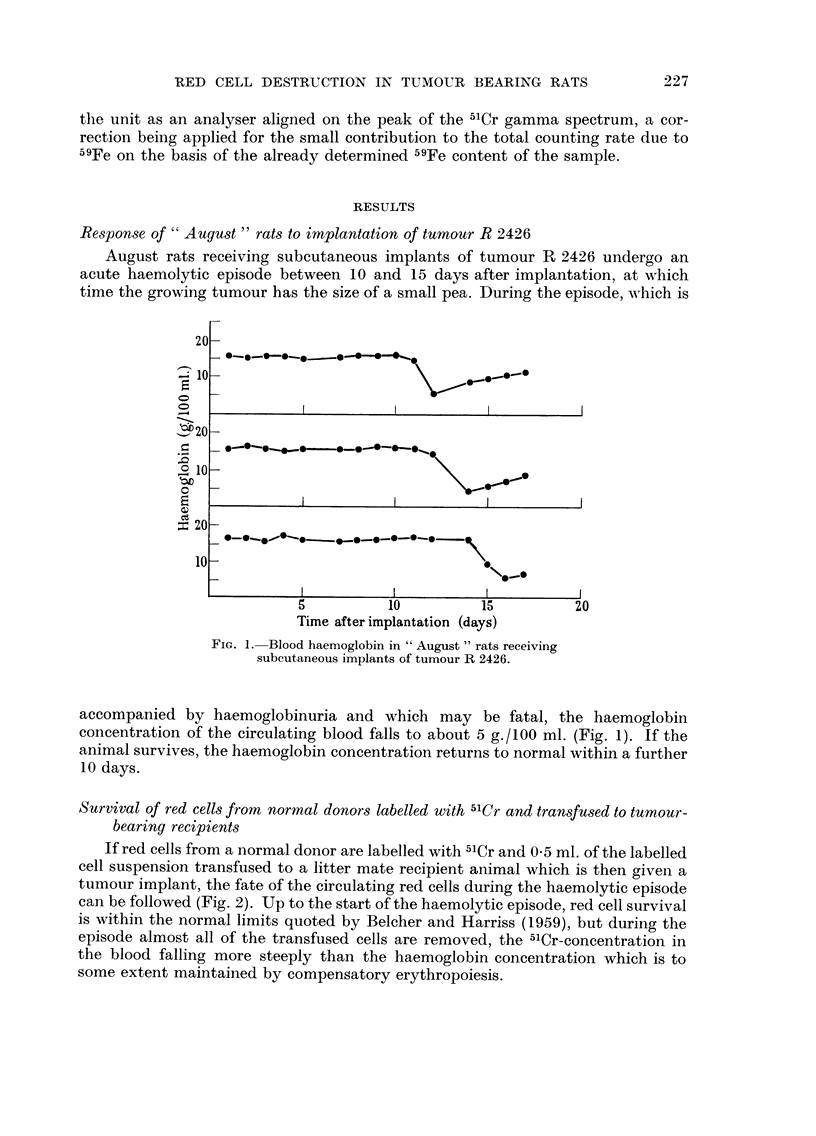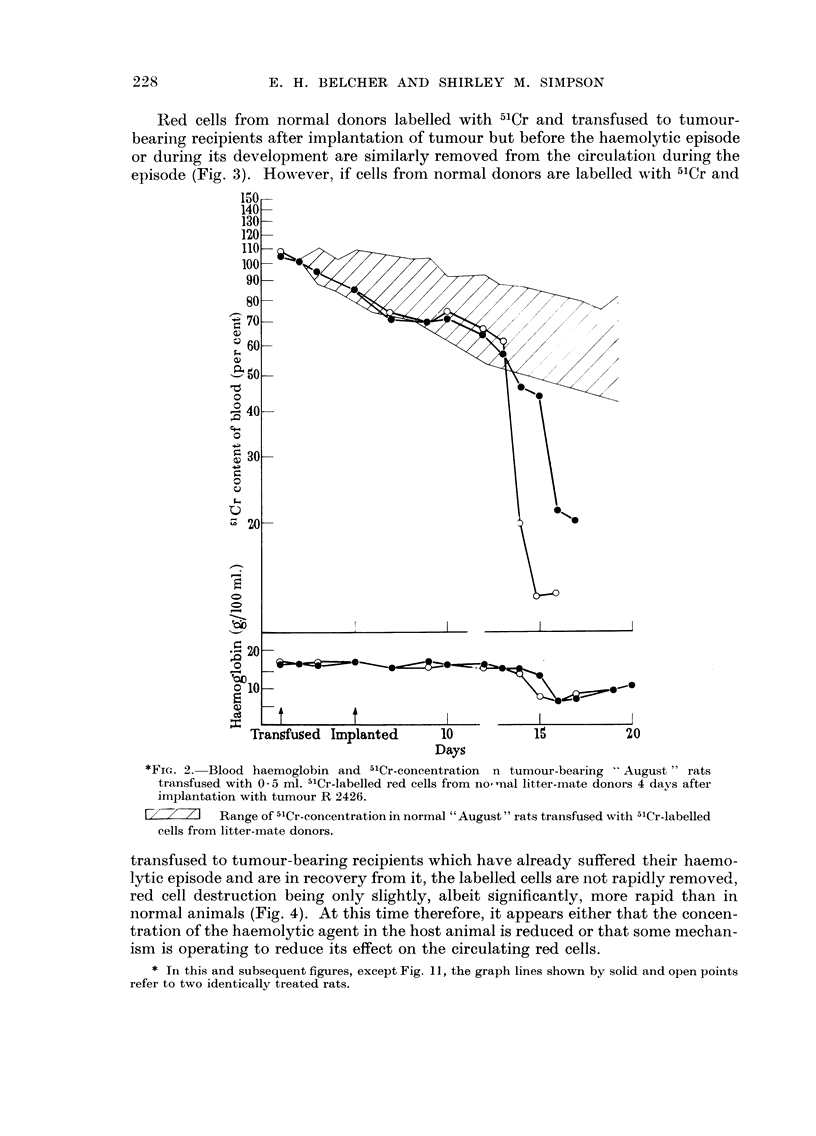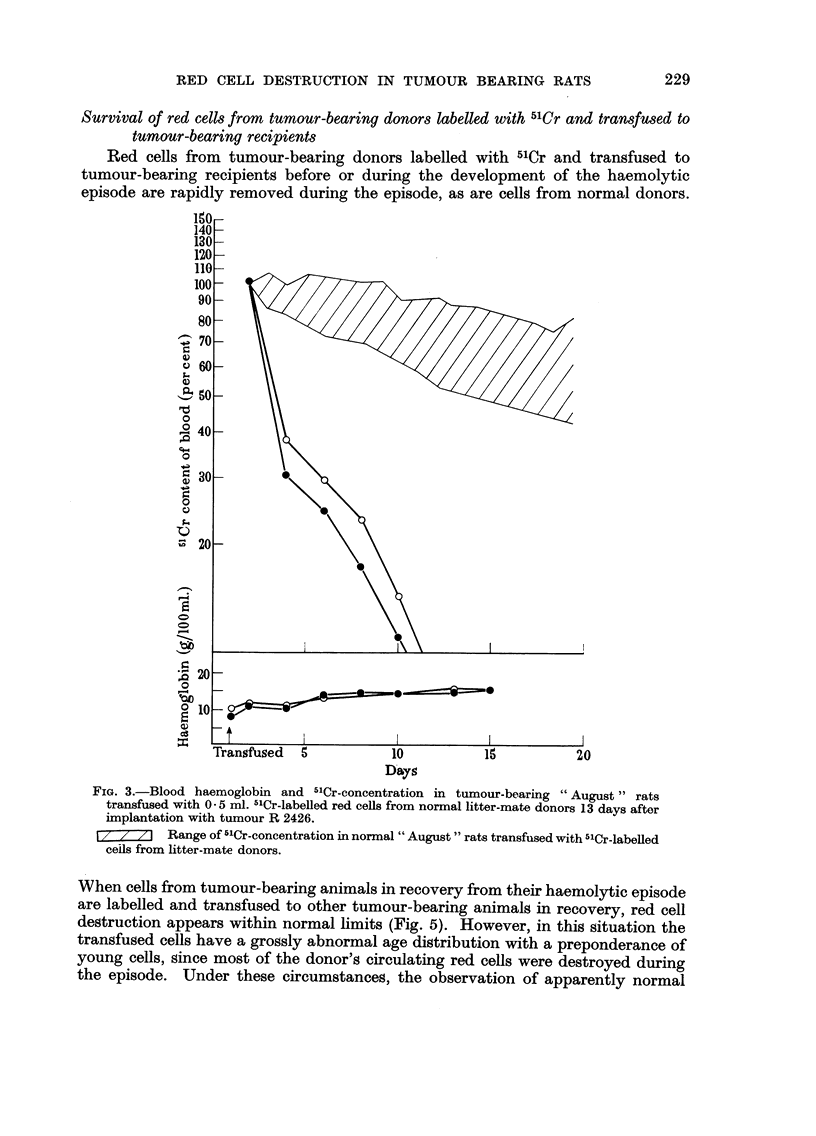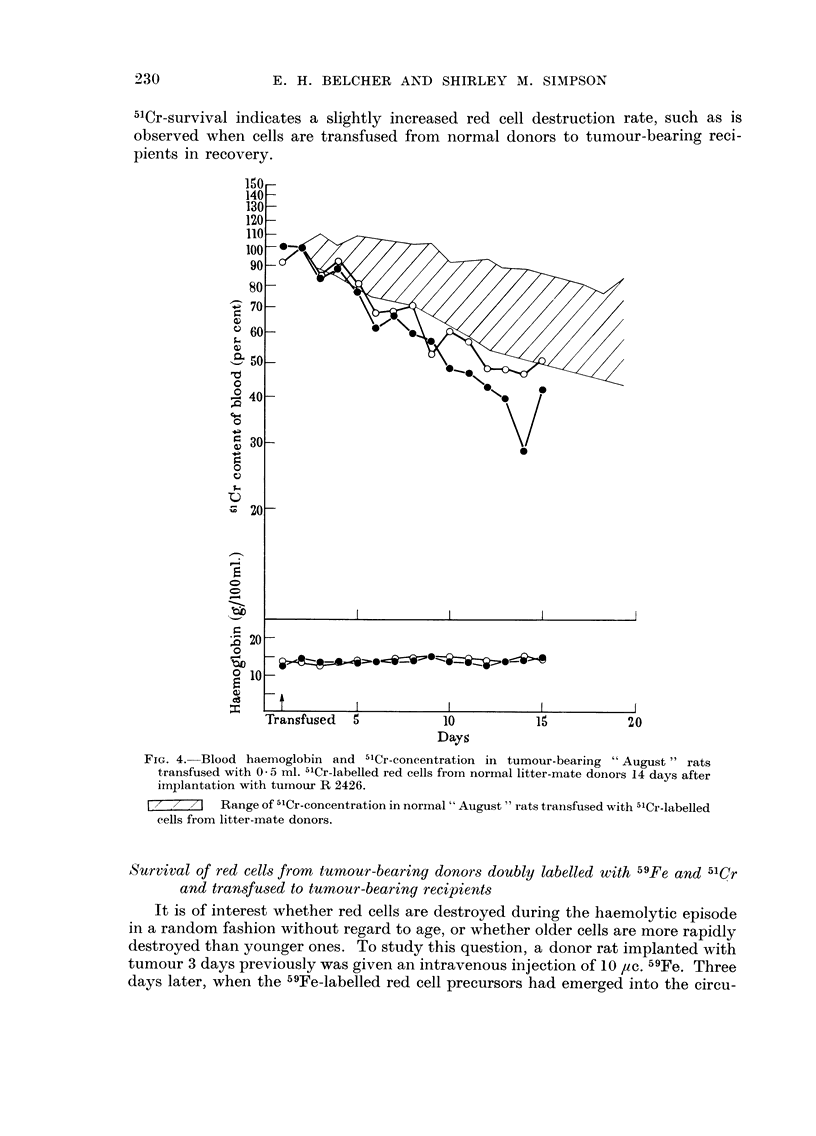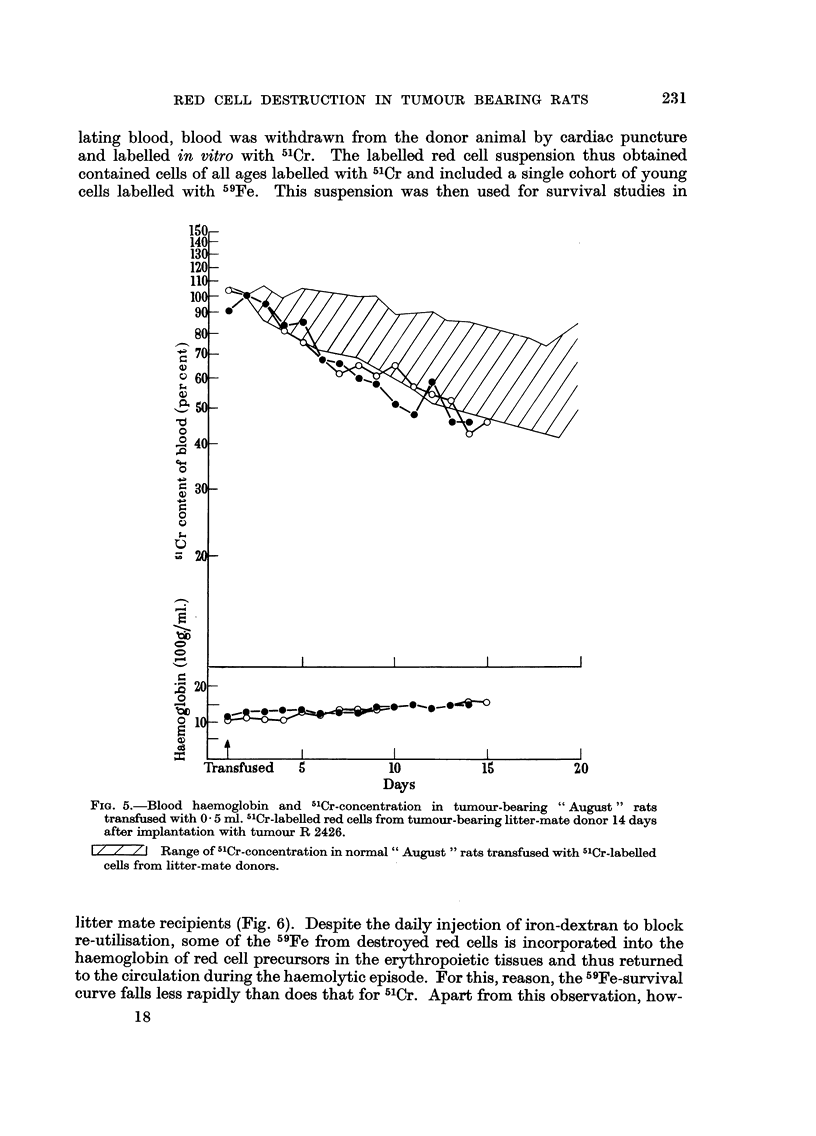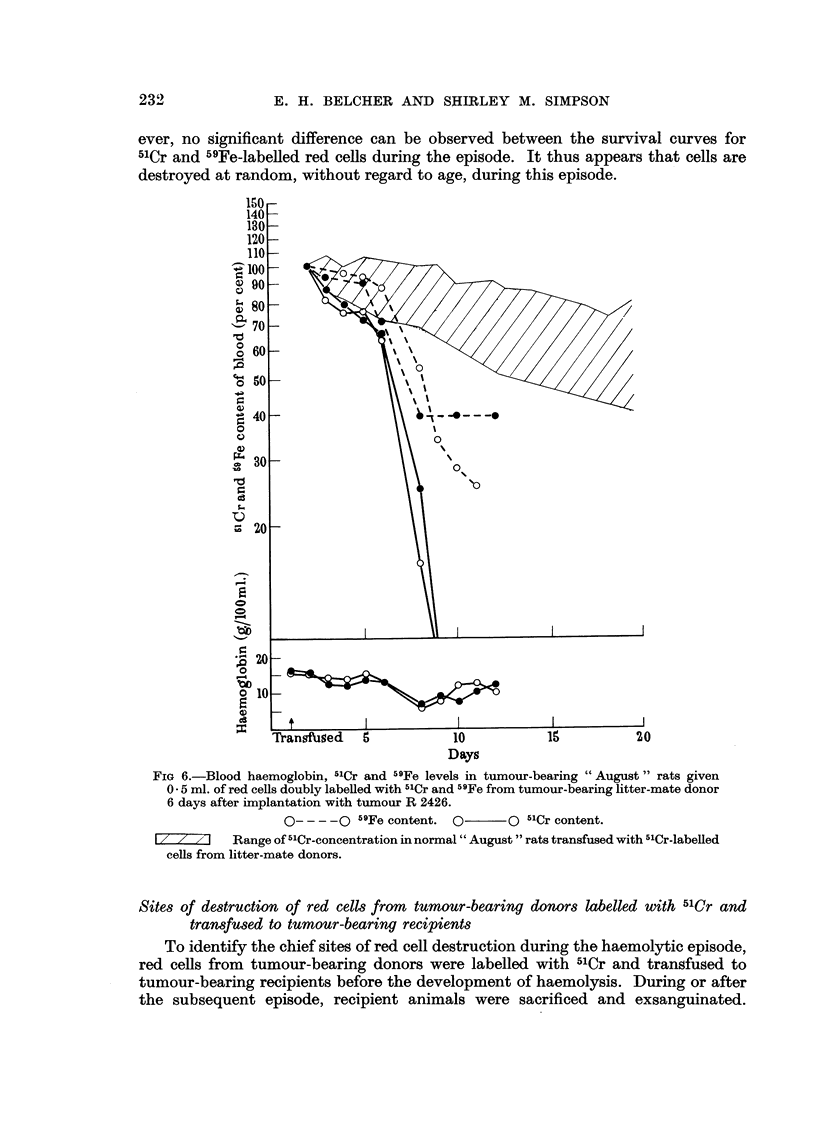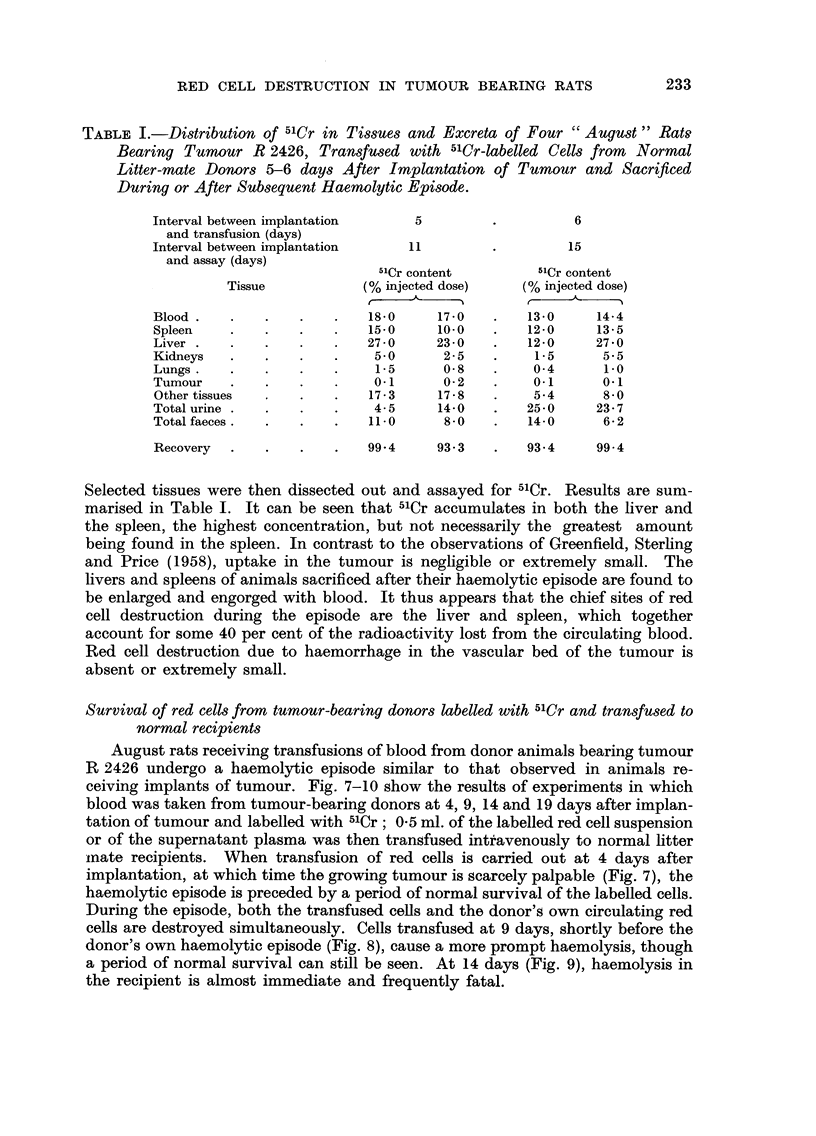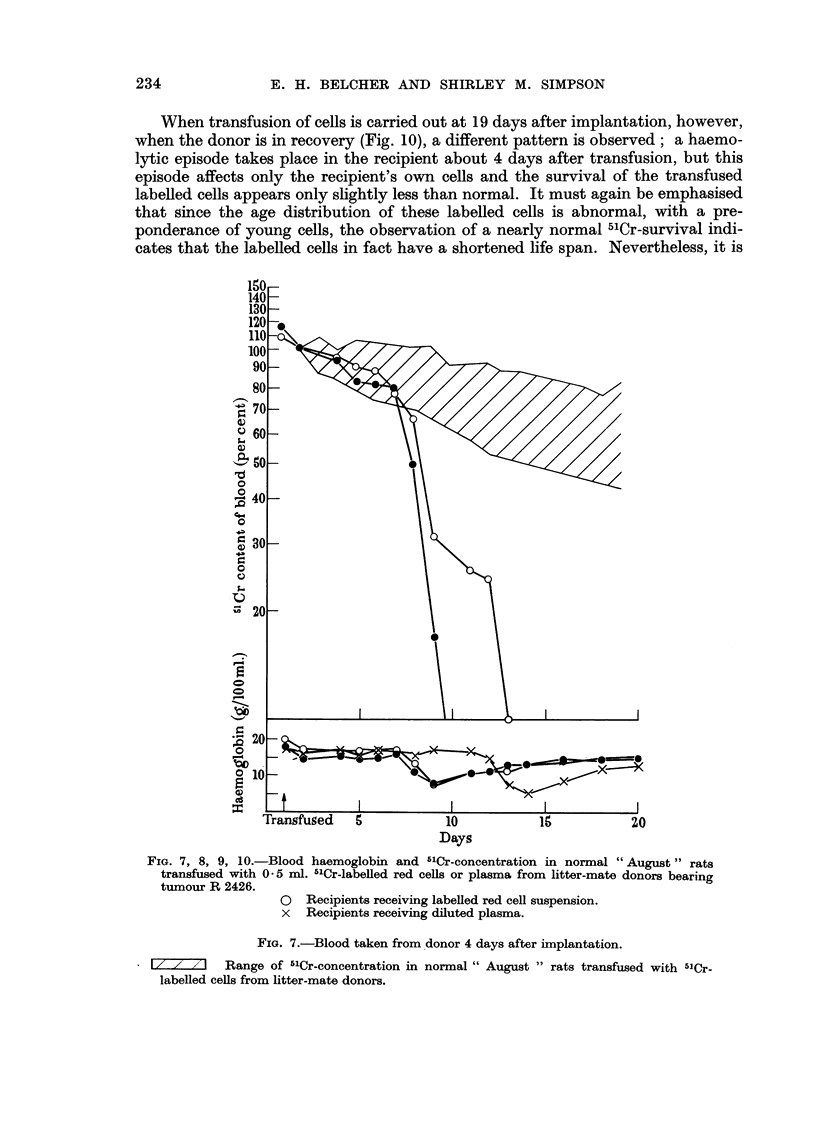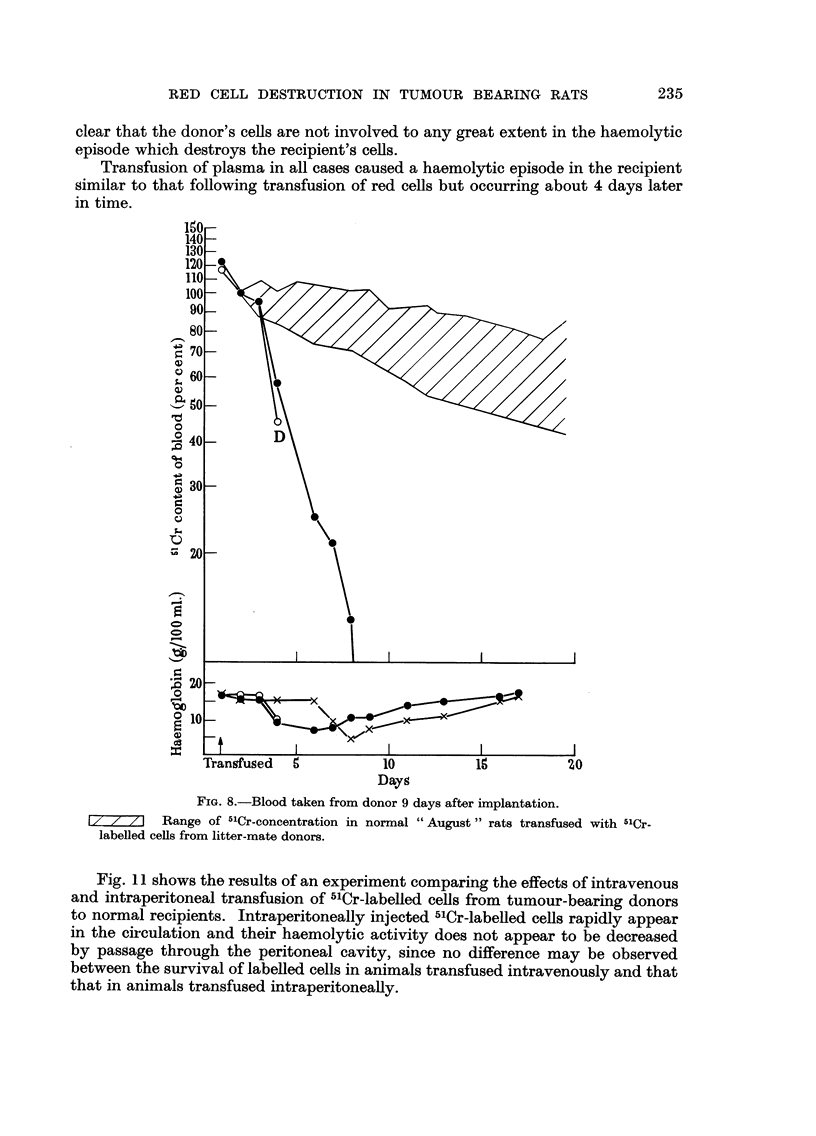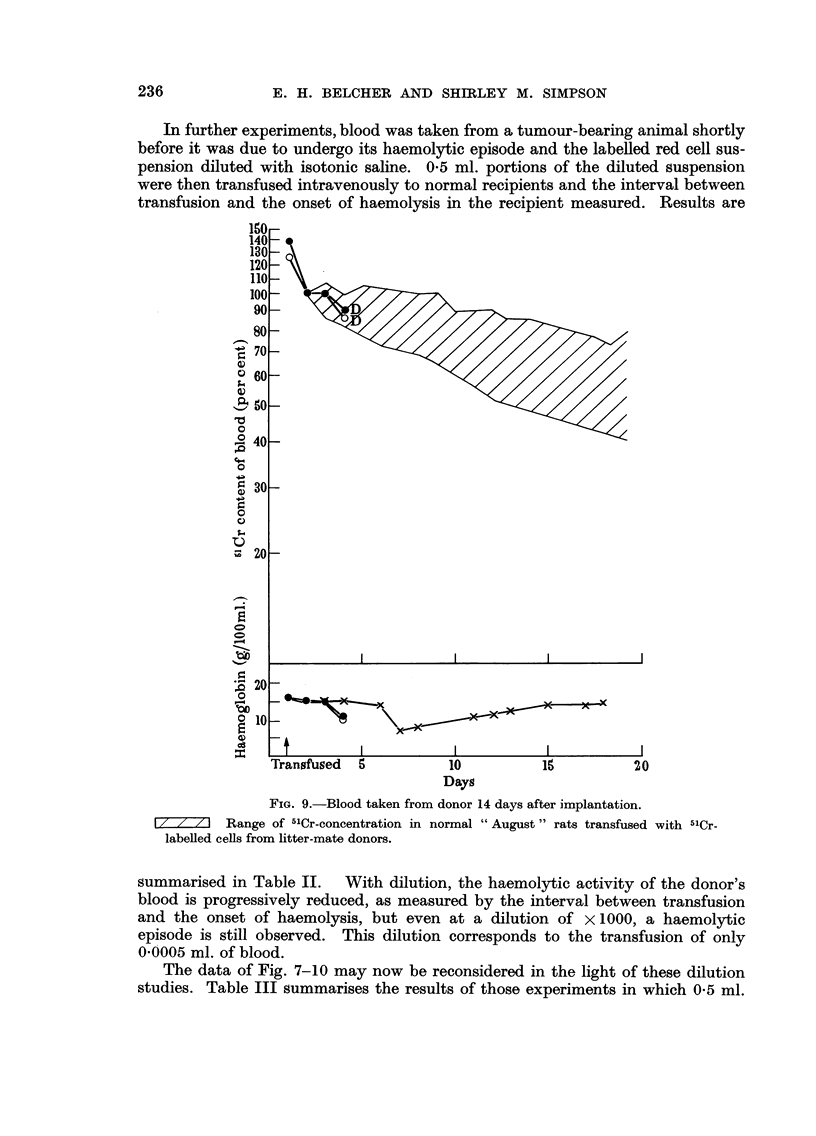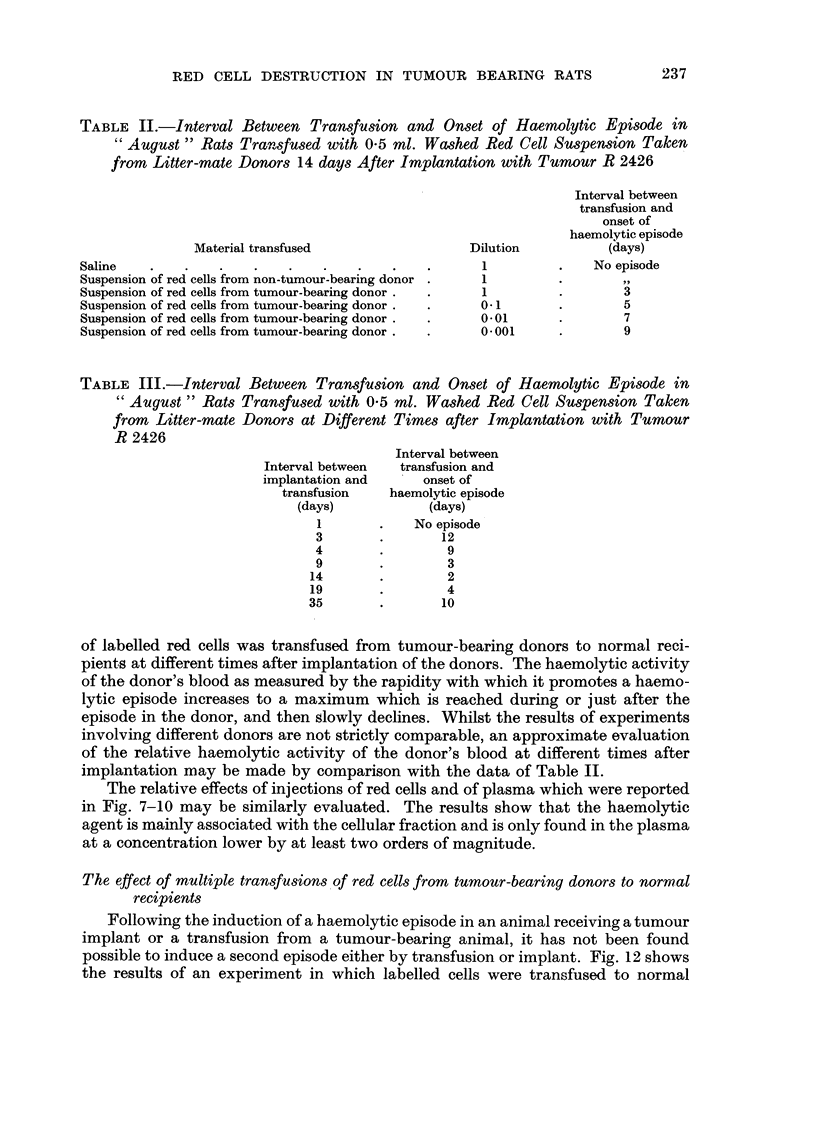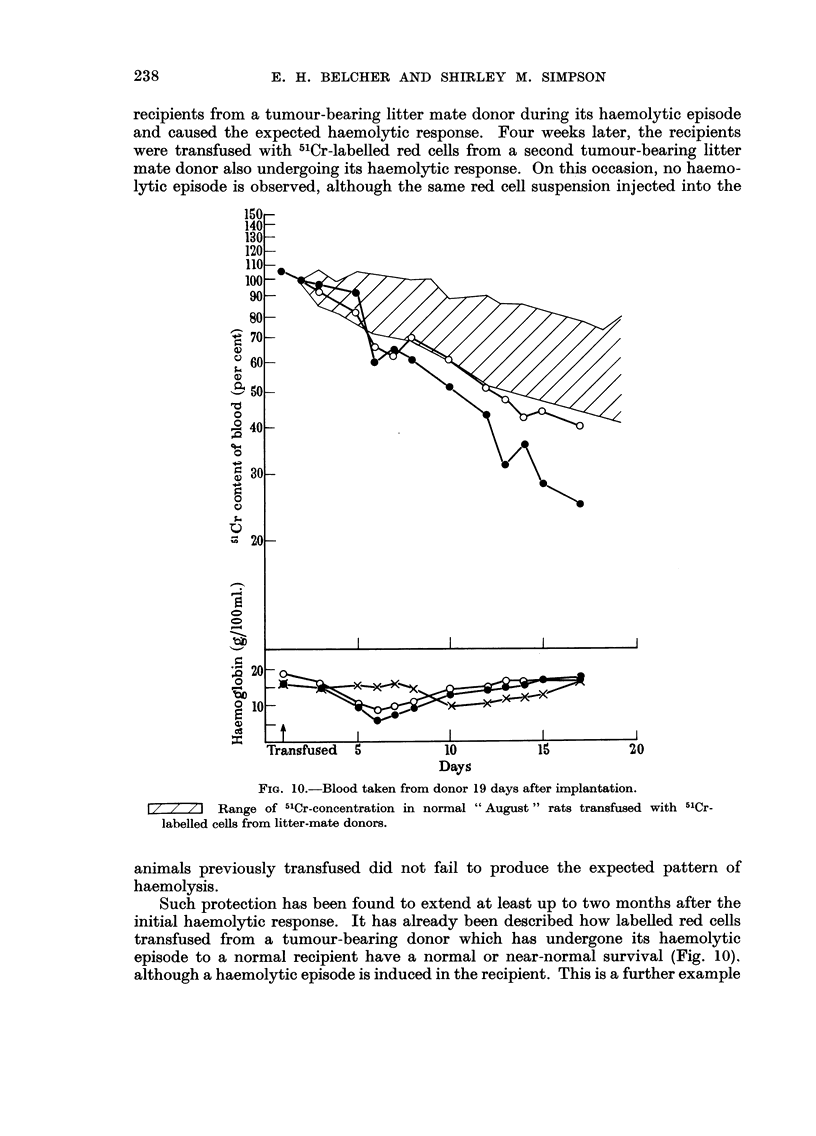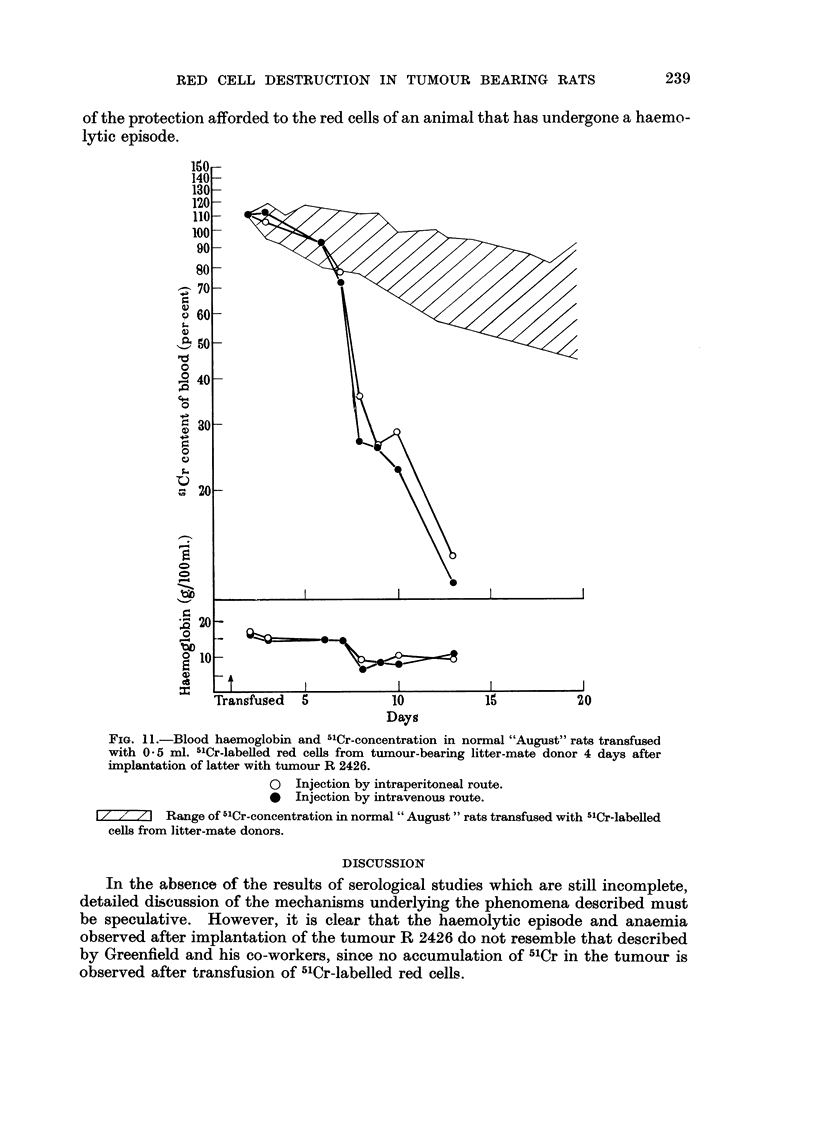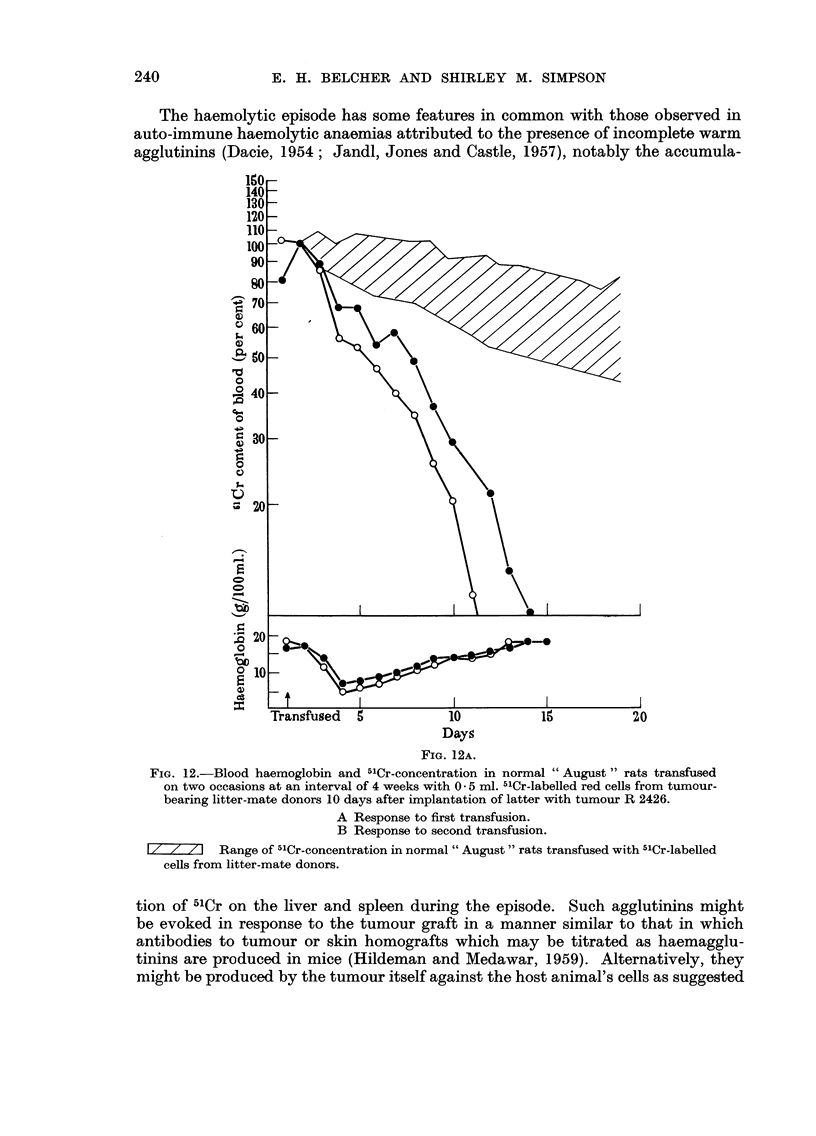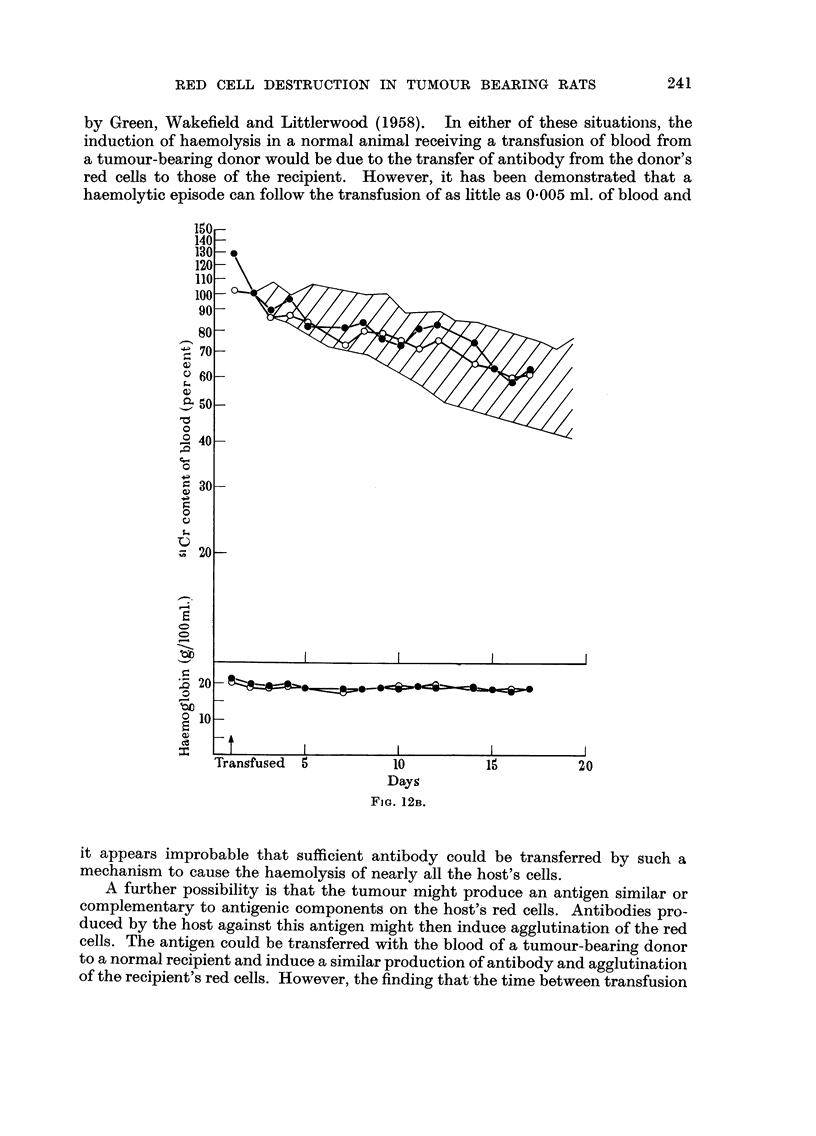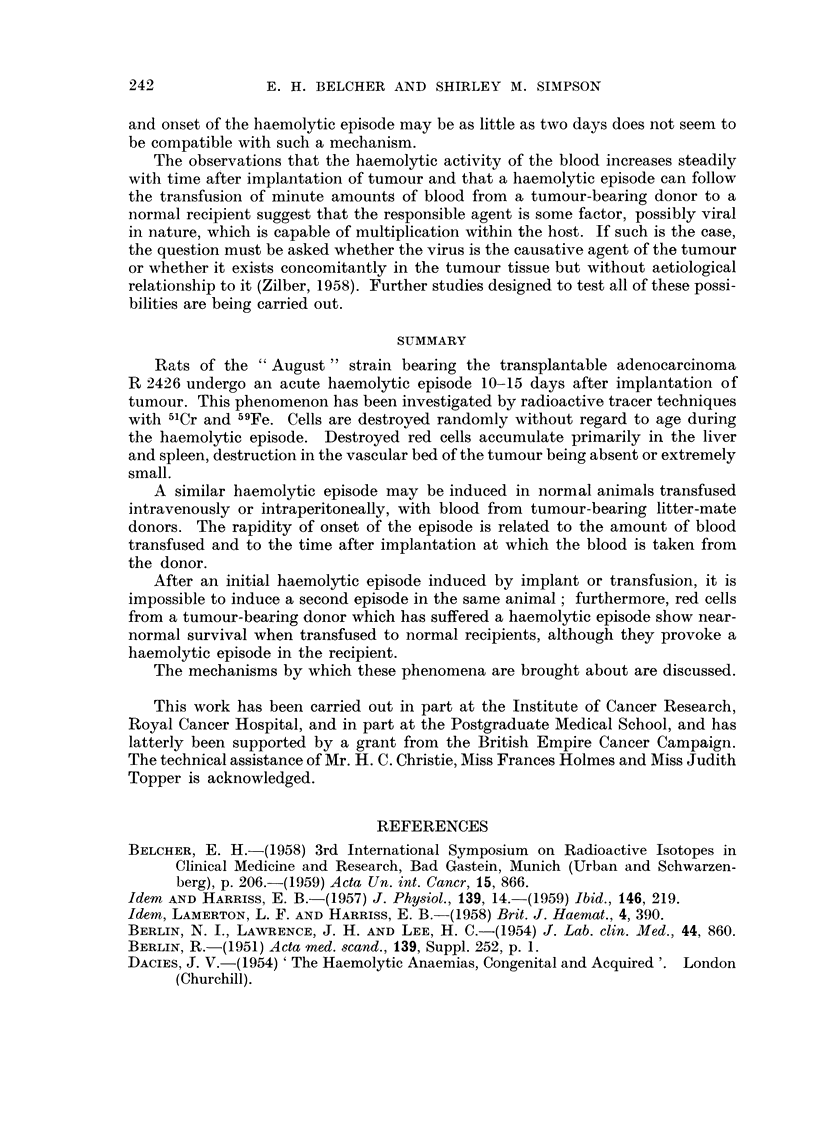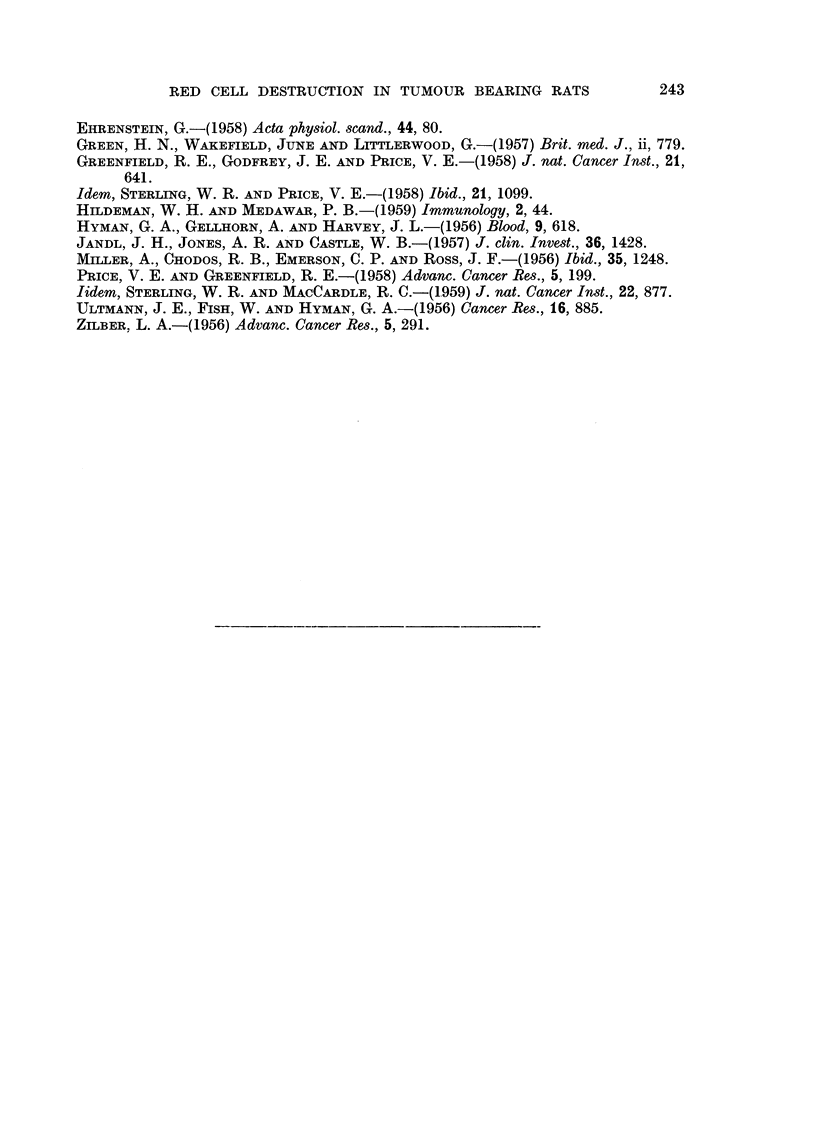# Radioactive Tracer Studies of Red Cell Destruction in Rats bearing a Transplantable Tumour

**DOI:** 10.1038/bjc.1960.28

**Published:** 1960-06

**Authors:** E. H. Belcher, Shirley M. Simpson


### RADIOACTIVE TRACER STUDIES OF RED CELL DESTRUCTION IN RATS BEARING A TRANSPLANTABLE TUMOUR

E. H. BELCHER AND SHIRLEY M. SIMPSON
*From the Postgraduate Medical School, London, W.12*

Received for publication April 2, 1960

ANAEMIA is frequently observed in cancer patients and also in animals bearing transplantable or induced tumours ; this association is the subject of a recent review by Price and Greenfield (1958). It is probable that both decreased erythropoiesis and increased erythrocyte destruction contribute to the reduction in haemoglobin concentration, haematocrit value or red cell volume observed in anaemias associated with cancer. However, evidence has accumulated that an increased rate of destruction of the circulating red cells is common in human cancer. Thus Berlin (1951) demonstrated by the Ashby differential agglutination technique an increased rate of destruction of transfused normal cells in patients with myeloid or lymphatic leukaemia. An increased rate of destruction of the patient's own circulating red cells labelled with glycine-2-$^{14}C$ was found by Berlin, Lawrence and Lee (1954) in leukaemia. Hyman, Gellhorn and Harvey (1956), using both the Ashby technique and the transfusion of cells labelled with $^{51}Cr$ found an increased rate of destruction of cells transfused from normal donors to patients with carcinomata and other neoplasms, whereas cells transfused from cancer patients to normal volunteers had a normal or near-normal life span. Hence they argued the absence of any intrinsic defect in the red cells of cancer patients. Miller, Chodos, Emerson and Ross (1956) also demonstrated by the Ashby and the $^{51}Cr$-techniques an increased rate of destruction of red cells transfused from normal subjects to patients with cancer of various sites or autotransfused to such patients.

The results of studies on animals bearing transplantable or induced tumours have been more conflicting. Ultman, Fish and Hyman (1956), in studies of $^{51}Cr$-survival in rabbits bearing the Brown-Pearce or XV2 carcinoma, failed to reveal any increased destruction of red cells except in animals with severe infection. Ehrenstein (1958) used glycine-2-$^{14}C$ to study the life span of the circulating red cells in mice bearing the Ehrlich ascites carcinoma and found increased red cell destruction of the random type in tumour-bearing animals. Greenfield, Godfrey and Price (1958), using $^{59}Fe$-labelled cells, also observed increased red cell destruction in rats bearing Lymphosarcoma 2788, Hepatoma 3683, the Yoshida ascites tumour and the Harderian gland Carcinoma 2226. They found that as red cell destruction proceeded, $^{59}Fe$ accumulated within the tumour. In all of these tumours, haemorrhage or thrombosis was observed. In the mouse Fibrosarcoma HE 8971 and Hepatoma 134 (ascites) and the rat Fibrosarcoma 4956, in which there was little or no haemorrhage or thrombosis, anaemia and loss of labelled red cells was small and did not occur until the late stages of tumour growth. In further studies by the same group, Greenfield, Sterling and Price (1958) transfused $^{51}Cr$-labelled red cells to rats bearing the Lymphosarcoma R 2788 and were able to recover from the tumour nearly all the $^{51}Cr$ lost from the blood ; they concluded that increased red cell destruction was the consequence of vascular injury in the neighbourhood of the tumour. Price, Greenfield, Sterling and MacCardle (1959) transfused mixtures of $^{51}Cr$- and $^{59}Fe$-labelled red cells to rats with Lymphosarcoma R 2788 and found that the subsequent distributions of the two isotopes within the tumour were similar, uptake being greatest in haemorrhagic areas, less in necrotic areas and almost negligible in white viable tumour. In this tumour, haemorrhage into the surrounding connective tissue is an important phenomenon. The extent of increased red cell destruction in other tumours could be correlated with their histological appearance. Thus in Fibrosarcoma 4956, little haemorrhage was observed and increased red cell destruction was not detected until the tumour had become massive and ulceration of the skin had occurred.

In the investigations described below, red cell destruction has been studied by the $^{51}Cr$- and $^{59}Fe$-techniques in rats of the " August " strain bearing the transplantable mammary adenocarcinoma R 2426, in which neither haemorrhage nor necrosis are observed. Particular interest was attached to this tumour since a haemolytic episode in the host animal had been observed to follow its implantation. Preliminary reports of these studies have already been published (Belcher, 1958, 1959).

### MATERIALS AND METHODS

*Rats.*—Rats of the pure-line " August " strain of weight 100–200 g. were used throughout. They were maintained on Medical Research Council Diet No. 41 and water *ad libitum.* Frequent observations on splenectomised animals failed to reveal any latent *Bartonella muris* infection.

*Transplantation of tumour.*—Tumour R 2426 was transplanted at intervals of about 4 weeks. Implantation of tumour was carried subcutaneously under aseptic conditions through a trochar inserted into the left abdominal wall, the site of implantation being afterwards closed by a small suture clip. Four weeks after implantation, when transplantation was again due, the growing tumour was firm and round with a maximum diameter of about 2 cm. On sectioning, it was normally found to be white and viable throughout, with no evidence of necrotic or haemorrhagic areas.

### *Studies with $^{51}Cr$-labelled red cells*

Red cell survival studies with $^{51}Cr$ were made as described by Belcher and Harriss (1959). In order to reduce the possibility of uncontrolled transfusion reactions, transfusion of blood was carried out under aseptic conditions between male litter mates, one member of each litter acting as donor, the others as recipients. In studies with $^{51}Cr$, the red cells of donor animals were labelled *in vitro* with $^{51}Cr$ as follows : 2 ml. of blood were withdrawn by cardiac puncture into a syringe containing 0·1 ml. heparin (" Pularin ", Evans Medical Supplies Ltd.), and delivered into a graduated test tube. One ml. of acid-citrate dextrose-solution and 1 ml. of a solution of sodium chromate—$^{51}Cr$ in isotonic saline containing about 50 $\mu$c./ml. $^{51}Cr$ (specific activity about 10 $\mu$c./$\mu$g. Cr) were added. The mixture was allowed to stand for 30 minutes with occasional shaking. It was then gently centrifuged, the supernatant diluted plasma pipetted off and the cells resuspended in isotonic saline, the final volume being readjusted to 2 ml. The labelled cell suspension was further diluted with isotonic saline if desired. Of the added $^{51}$Cr, 90–95 per cent entered the red cells.

Portions of the labelled cell suspension 0·5 ml. in volume were injected without delay into litter mate recipients, injection being carried out either intravenously into a lateral tail vein or intraperitoneally. At daily intervals thereafter, the extreme tip of the tail of each recipient was cut under light ether anaethesia and a 0·02 ml. blood sample taken into a haemoglobin pipette and delivered into 5 ml. ammoniated distilled water for haemoglobin estimation. Haemoglobin was estimated colorimetrically as $HbO_2$ in the haemolysed blood samples. These were then transferred to $\frac{5}{8}$ inch diameter sample tubes and assayed for $^{51}$Cr in a well scintillation counter coupled to a scaler through a linear amplifier and single channel pulse height discriminator/analyser. The $^{51}$Cr concentrations in successive samples of blood were expressed as percentages of that of the sample taken from the same animal on the first day after transfusion and the result corrected for the increase in blood volume with increase in weight of the animal during the period of observation (Belcher and Harriss, 1957).

Studies of $^{51}$Cr-distribution in the tissues were made as described by Belcher, Lamerton and Harriss (1958). Recipient animals were killed by cervical fracture under ether anaesthesia. They were then exsanguinated as completely as possible by opening the thorax, excising the left lung and injecting isotonic saline into the left ventricle of the heart, diluted blood being removed as it accumulated in the thoracic cavity. The carcasses were then carefully dissected and selected tissues removed to $\frac{5}{8}$ inch diameter sample tubes for $^{51}$Cr-assay. Small corrections were applied for the variation in efficiency of the scintillation counter with sample volume.

*Studies with $^{59}$Fe-labelled red cells*

Red cell survival studies with $^{59}$Fe were made as described by Belcher and Harriss, 1959. The circulating red cells of donor animals were labelled *in vivo* with $^{59}$Fe as follows ; 10 $\mu$c. $^{59}$Fe (specific activity 1–5 $\mu$c./$\mu$g. Fe) as ferric chloride in 0·5 ml. 1 per cent w/v sodium citrate solution were injected intravenously to each donor. After an interval of 3 days to allow cells labelled in the erythropoietic tissues to emerge into the circulation, about 2 ml. of blood were withdrawn by cardiac puncture from each donor into a heparinised syringe and delivered into a graduated test tube. The $^{59}$Fe-labelled red cells were further labelled *in vitro* with $^{51}$Cr as described above if desired. Portions of labelled cell suspension 0·5 ml. in volume were then injected intravenously into litter mate recipients. Daily 0·02 ml. blood samples were taken from the cut tails of the recipient animals as already described, haemolysed in 5 ml. ammoniated distilled water and transferred to $\frac{5}{8}$ inch diameter sample tubes for radioactive assay. To suppress re-utilisation of $^{59}$Fe from destroyed red cells by the erythropoietic tissues daily injections of 0·1 ml./100 g. body weight of an iron-dextran complex (" Imferon ", Bengers Laboratories Ltd.) containing 50 mg./ml. Fe were given intramuscularly throughout studies of red cell survival of $^{59}$Fe-labelled cells.

In the radioactive assay of samples in single tracer experiments with $^{51}$Cr or $^{59}$Fe alone, the discriminator/analyser unit was operated as a simple discriminator. In double tracer experiments with $^{51}$Cr and $^{59}$Fe, samples were first assayed for $^{59}$Fe by using the unit as a discriminator set at a level such that the system was almost completely insensitive to $^{51}$Cr. Samples were then assayed for $^{51}$Cr using the unit as an analyser aligned on the peak of the $^{51}Cr$ gamma spectrum, a correction being applied for the small contribution to the total counting rate due to $^{59}Fe$ on the basis of the already determined $^{59}Fe$ content of the sample.

### RESULTS

*Response of " August " rats to implantation of tumour R* 2426

August rats receiving subcutaneous implants of tumour R 2426 undergo an acute haemolytic episode between 10 and 15 days after implantation, at which time the growing tumour has the size of a small pea. During the episode, which is

FIG. 1.—Blood haemoglobin in " August " rats receiving subcutaneous implants of tumour R 2426.

accompanied by haemoglobinuria and which may be fatal, the haemoglobin concentration of the circulating blood falls to about 5 g./100 ml. (Fig. 1). If the animal survives, the haemoglobin concentration returns to normal within a further 10 days.

*Survival of red cells from normal donors labelled with $^{51}Cr$ and transfused to tumour-bearing recipients*

If red cells from a normal donor are labelled with $^{51}Cr$ and 0·5 ml. of the labelled cell suspension transfused to a litter mate recipient animal which is then given a tumour implant, the fate of the circulating red cells during the haemolytic episode can be followed (Fig. 2). Up to the start of the haemolytic episode, red cell survival is within the normal limits quoted by Belcher and Harriss (1959), but during the episode almost all of the transfused cells are removed, the $^{51}Cr$-concentration in the blood falling more steeply than the haemoglobin concentration which is to some extent maintained by compensatory erythropoiesis.

Red cells from normal donors labelled with $^{51}Cr$ and transfused to tumour-bearing recipients after implantation of tumour but before the haemolytic episode or during its development are similarly removed from the circulation during the episode (Fig. 3). However, if cells from normal donors are labelled with $^{51}Cr$ and

*Fig. 2.—Blood haemoglobin and $^{51}Cr$-concentration n tumour-bearing "August" rats transfused with 0·5 ml. $^{51}Cr$-labelled red cells from no' mal litter-mate donors 4 days after implantation with tumour R 2426.

Range of $^{51}Cr$-concentration in normal "August" rats transfused with $^{51}Cr$-labelled cells from litter-mate donors.

transfused to tumour-bearing recipients which have already suffered their haemolytic episode and are in recovery from it, the labelled cells are not rapidly removed, red cell destruction being only slightly, albeit significantly, more rapid than in normal animals (Fig. 4). At this time therefore, it appears either that the concentration of the haemolytic agent in the host animal is reduced or that some mechanism is operating to reduce its effect on the circulating red cells.

* In this and subsequent figures, except Fig. 11, the graph lines shown by solid and open points refer to two identically treated rats.

*Survival of red cells from tumour-bearing donors labelled with $^{51}Cr$ and transfused to tumour-bearing recipients*

Red cells from tumour-bearing donors labelled with $^{51}$Cr and transfused to tumour-bearing recipients before or during the development of the haemolytic episode are rapidly removed during the episode, as are cells from normal donors.

FIG. 3.—Blood haemoglobin and $^{51}$Cr-concentration in tumour-bearing "August" rats transfused with 0·5 ml. $^{51}$Cr-labelled red cells from normal litter-mate donors 13 days after implantation with tumour R 2426.

Range of $^{51}$Cr-concentration in normal "August" rats transfused with $^{51}$Cr-labelled cells from litter-mate donors.

When cells from tumour-bearing animals in recovery from their haemolytic episode are labelled and transfused to other tumour-bearing animals in recovery, red cell destruction appears within normal limits (Fig. 5). However, in this situation the transfused cells have a grossly abnormal age distribution with a preponderance of young cells, since most of the donor's circulating red cells were destroyed during the episode. Under these circumstances, the observation of apparently normal

$^{51}$Cr-survival indicates a slightly increased red cell destruction rate, such as is observed when cells are transfused from normal donors to tumour-bearing recipients in recovery.

Fig. 4.—Blood haemoglobin and $^{51}$Cr-concentration in tumour-bearing "August" rats transfused with 0·5 ml. $^{51}$Cr-labelled red cells from normal litter-mate donors 14 days after implantation with tumour R 2426.

[hatched box] Range of $^{51}$Cr-concentration in normal "August" rats transfused with $^{51}$Cr-labelled cells from litter-mate donors.

*Survival of red cells from tumour-bearing donors doubly labelled with $^{59}$Fe and $^{51}$Cr and transfused to tumour-bearing recipients*

It is of interest whether red cells are destroyed during the haemolytic episode in a random fashion without regard to age, or whether older cells are more rapidly destroyed than younger ones. To study this question, a donor rat implanted with tumour 3 days previously was given an intravenous injection of 10 $\mu$c. $^{59}$Fe. Three days later, when the $^{59}$Fe-labelled red cell precursors had emerged into the circulating blood, blood was withdrawn from the donor animal by cardiac puncture and labelled *in vitro* with $^{51}Cr$. The labelled red cell suspension thus obtained contained cells of all ages labelled with $^{51}Cr$ and included a single cohort of young cells labelled with $^{59}Fe$. This suspension was then used for survival studies in

FIG. 5.—Blood haemoglobin and $^{51}Cr$-concentration in tumour-bearing "August" rats transfused with 0·5 ml. $^{51}Cr$-labelled red cells from tumour-bearing litter-mate donor 14 days after implantation with tumour R 2426.

▨ Range of $^{51}Cr$-concentration in normal "August" rats transfused with $^{51}Cr$-labelled cells from litter-mate donors.

litter mate recipients (Fig. 6). Despite the daily injection of iron-dextran to block re-utilisation, some of the $^{59}Fe$ from destroyed red cells is incorporated into the haemoglobin of red cell precursors in the erythropoietic tissues and thus returned to the circulation during the haemolytic episode. For this, reason, the $^{59}Fe$-survival curve falls less rapidly than does that for $^{51}Cr$. Apart from this observation, however, no significant difference can be observed between the survival curves for $^{51}Cr$ and $^{59}Fe$-labelled red cells during the episode. It thus appears that cells are destroyed at random, without regard to age, during this episode.

FIG 6.—Blood haemoglobin, $^{51}Cr$ and $^{59}Fe$ levels in tumour-bearing " August " rats given 0·5 ml. of red cells doubly labelled with $^{51}Cr$ and $^{59}Fe$ from tumour-bearing litter-mate donor 6 days after implantation with tumour R 2426.

○- - - -○ $^{59}Fe$ content. ○———○ $^{51}Cr$ content.

▨ Range of $^{51}Cr$-concentration in normal " August " rats transfused with $^{51}Cr$-labelled cells from litter-mate donors.

*Sites of destruction of red cells from tumour-bearing donors labelled with $^{51}Cr$ and transfused to tumour-bearing recipients*

To identify the chief sites of red cell destruction during the haemolytic episode, red cells from tumour-bearing donors were labelled with $^{51}Cr$ and transfused to tumour-bearing recipients before the development of haemolysis. During or after the subsequent episode, recipient animals were sacrificed and exsanguinated.

TABLE I.—*Distribution of $^{51}Cr$ in Tissues and Excreta of Four "August" Rats Bearing Tumour R 2426, Transfused with $^{51}Cr$-labelled Cells from Normal Litter-mate Donors 5–6 days After Implantation of Tumour and Sacrificed During or After Subsequent Haemolytic Episode.*

| | | | |
|---|---|---|---|
| Interval between implantation and transfusion (days) | 5 | | 6 | |
| Interval between implantation and assay (days) | 11 | | 15 | |
| Tissue | $^{51}$Cr content (% injected dose) | | $^{51}$Cr content (% injected dose) | |
| Blood | 18·0 | 17·0 | 13·0 | 14·4 |
| Spleen | 15·0 | 10·0 | 12·0 | 13·5 |
| Liver | 27·0 | 23·0 | 12·0 | 27·0 |
| Kidneys | 5·0 | 2·5 | 1·5 | 5·5 |
| Lungs | 1·5 | 0·8 | 0·4 | 1·0 |
| Tumour | 0·1 | 0·2 | 0·1 | 0·1 |
| Other tissues | 17·3 | 17·8 | 5·4 | 8·0 |
| Total urine | 4·5 | 14·0 | 25·0 | 23·7 |
| Total faeces | 11·0 | 8·0 | 14·0 | 6·2 |
| Recovery | 99·4 | 93·3 | 93·4 | 99·4 |

Selected tissues were then dissected out and assayed for $^{51}$Cr. Results are summarised in Table I. It can be seen that $^{51}$Cr accumulates in both the liver and the spleen, the highest concentration, but not necessarily the greatest amount being found in the spleen. In contrast to the observations of Greenfield, Sterling and Price (1958), uptake in the tumour is negligible or extremely small. The livers and spleens of animals sacrificed after their haemolytic episode are found to be enlarged and engorged with blood. It thus appears that the chief sites of red cell destruction during the episode are the liver and spleen, which together account for some 40 per cent of the radioactivity lost from the circulating blood. Red cell destruction due to haemorrhage in the vascular bed of the tumour is absent or extremely small.

*Survival of red cells from tumour-bearing donors labelled with $^{51}Cr$ and transfused to normal recipients*

August rats receiving transfusions of blood from donor animals bearing tumour R 2426 undergo a haemolytic episode similar to that observed in animals receiving implants of tumour. Fig. 7–10 show the results of experiments in which blood was taken from tumour-bearing donors at 4, 9, 14 and 19 days after implantation of tumour and labelled with $^{51}$Cr ; 0·5 ml. of the labelled red cell suspension or of the supernatant plasma was then transfused intravenously to normal litter mate recipients. When transfusion of red cells is carried out at 4 days after implantation, at which time the growing tumour is scarcely palpable (Fig. 7), the haemolytic episode is preceded by a period of normal survival of the labelled cells. During the episode, both the transfused cells and the donor's own circulating red cells are destroyed simultaneously. Cells transfused at 9 days, shortly before the donor's own haemolytic episode (Fig. 8), cause a more prompt haemolysis, though a period of normal survival can still be seen. At 14 days (Fig. 9), haemolysis in the recipient is almost immediate and frequently fatal.

When transfusion of cells is carried out at 19 days after implantation, however, when the donor is in recovery (Fig. 10), a different pattern is observed ; a haemolytic episode takes place in the recipient about 4 days after transfusion, but this episode affects only the recipient's own cells and the survival of the transfused labelled cells appears only slightly less than normal. It must again be emphasised that since the age distribution of these labelled cells is abnormal, with a preponderance of young cells, the observation of a nearly normal $^{51}$Cr-survival indicates that the labelled cells in fact have a shortened life span. Nevertheless, it is

FIG. 7, 8, 9, 10.—Blood haemoglobin and $^{51}$Cr-concentration in normal " August " rats transfused with 0·5 ml. $^{51}$Cr-labelled red cells or plasma from litter-mate donors bearing tumour R 2426.

○ Recipients receiving labelled red cell suspension.
× Recipients receiving diluted plasma.

FIG. 7.—Blood taken from donor 4 days after implantation.

▨ Range of $^{51}$Cr-concentration in normal " August " rats transfused with $^{51}$Cr-labelled cells from litter-mate donors.

clear that the donor's cells are not involved to any great extent in the haemolytic episode which destroys the recipient's cells.

Transfusion of plasma in all cases caused a haemolytic episode in the recipient similar to that following transfusion of red cells but occurring about 4 days later in time.

FIG. 8.—Blood taken from donor 9 days after implantation.

Range of $^{51}$Cr-concentration in normal " August " rats transfused with $^{51}$Cr-labelled cells from litter-mate donors.

Fig. 11 shows the results of an experiment comparing the effects of intravenous and intraperitoneal transfusion of $^{51}$Cr-labelled cells from tumour-bearing donors to normal recipients. Intraperitoneally injected $^{51}$Cr-labelled cells rapidly appear in the circulation and their haemolytic activity does not appear to be decreased by passage through the peritoneal cavity, since no difference may be observed between the survival of labelled cells in animals transfused intravenously and that that in animals transfused intraperitoneally.

In further experiments, blood was taken from a tumour-bearing animal shortly before it was due to undergo its haemolytic episode and the labelled red cell suspension diluted with isotonic saline. 0·5 ml. portions of the diluted suspension were then transfused intravenously to normal recipients and the interval between transfusion and the onset of haemolysis in the recipient measured. Results are

FIG. 9.—Blood taken from donor 14 days after implantation.

Range of $^{51}$Cr-concentration in normal " August " rats transfused with $^{51}$Cr-labelled cells from litter-mate donors.

summarised in Table II. With dilution, the haemolytic activity of the donor's blood is progressively reduced, as measured by the interval between transfusion and the onset of haemolysis, but even at a dilution of $\times 1000$, a haemolytic episode is still observed. This dilution corresponds to the transfusion of only 0·0005 ml. of blood.

The data of Fig. 7–10 may now be reconsidered in the light of these dilution studies. Table III summarises the results of those experiments in which 0·5 ml.

TABLE II.—*Interval Between Transfusion and Onset of Haemolytic Episode in "August" Rats Transfused with 0·5 ml. Washed Red Cell Suspension Taken from Litter-mate Donors 14 days After Implantation with Tumour R 2426*

| Material transfused | Dilution | Interval between transfusion and onset of haemolytic episode (days) |
|---|---|---|
| Saline | 1 | No episode |
| Suspension of red cells from non-tumour-bearing donor | 1 | ,, |
| Suspension of red cells from tumour-bearing donor | 1 | 3 |
| Suspension of red cells from tumour-bearing donor | 0·1 | 5 |
| Suspension of red cells from tumour-bearing donor | 0·01 | 7 |
| Suspension of red cells from tumour-bearing donor | 0·001 | 9 |

TABLE III.—*Interval Between Transfusion and Onset of Haemolytic Episode in "August" Rats Transfused with 0·5 ml. Washed Red Cell Suspension Taken from Litter-mate Donors at Different Times after Implantation with Tumour R 2426*

| Interval between implantation and transfusion (days) | Interval between transfusion and onset of haemolytic episode (days) |
|---|---|
| 1 | No episode |
| 3 | 12 |
| 4 | 9 |
| 9 | 3 |
| 14 | 2 |
| 19 | 4 |
| 35 | 10 |

of labelled red cells was transfused from tumour-bearing donors to normal recipients at different times after implantation of the donors. The haemolytic activity of the donor's blood as measured by the rapidity with which it promotes a haemolytic episode increases to a maximum which is reached during or just after the episode in the donor, and then slowly declines. Whilst the results of experiments involving different donors are not strictly comparable, an approximate evaluation of the relative haemolytic activity of the donor's blood at different times after implantation may be made by comparison with the data of Table II.

The relative effects of injections of red cells and of plasma which were reported in Fig. 7–10 may be similarly evaluated. The results show that the haemolytic agent is mainly associated with the cellular fraction and is only found in the plasma at a concentration lower by at least two orders of magnitude.

*The effect of multiple transfusions of red cells from tumour-bearing donors to normal recipients*

Following the induction of a haemolytic episode in an animal receiving a tumour implant or a transfusion from a tumour-bearing animal, it has not been found possible to induce a second episode either by transfusion or implant. Fig. 12 shows the results of an experiment in which labelled cells were transfused to normal recipients from a tumour-bearing litter mate donor during its haemolytic episode and caused the expected haemolytic response. Four weeks later, the recipients were transfused with $^{51}$Cr-labelled red cells from a second tumour-bearing litter mate donor also undergoing its haemolytic response. On this occasion, no haemolytic episode is observed, although the same red cell suspension injected into the

Fig. 10.—Blood taken from donor 19 days after implantation.

Range of $^{51}$Cr-concentration in normal "August" rats transfused with $^{51}$Cr-labelled cells from litter-mate donors.

animals previously transfused did not fail to produce the expected pattern of haemolysis.

Such protection has been found to extend at least up to two months after the initial haemolytic response. It has already been described how labelled red cells transfused from a tumour-bearing donor which has undergone its haemolytic episode to a normal recipient have a normal or near-normal survival (Fig. 10), although a haemolytic episode is induced in the recipient. This is a further example of the protection afforded to the red cells of an animal that has undergone a haemolytic episode.

FIG. 11.—Blood haemoglobin and $^{51}$Cr-concentration in normal "August" rats transfused with 0·5 ml. $^{51}$Cr-labelled red cells from tumour-bearing litter-mate donor 4 days after implantation of latter with tumour R 2426.

○ Injection by intraperitoneal route.
● Injection by intravenous route.

▨ Range of $^{51}$Cr-concentration in normal " August " rats transfused with $^{51}$Cr-labelled cells from litter-mate donors.

### DISCUSSION

In the absence of the results of serological studies which are still incomplete, detailed discussion of the mechanisms underlying the phenomena described must be speculative. However, it is clear that the haemolytic episode and anaemia observed after implantation of the tumour R 2426 do not resemble that described by Greenfield and his co-workers, since no accumulation of $^{51}$Cr in the tumour is observed after transfusion of $^{51}$Cr-labelled red cells.

The haemolytic episode has some features in common with those observed in auto-immune haemolytic anaemias attributed to the presence of incomplete warm agglutinins (Dacie, 1954 ; Jandl, Jones and Castle, 1957), notably the accumula-

FIG. 12A.

FIG. 12.—Blood haemoglobin and $^{51}$Cr-concentration in normal " August " rats transfused on two occasions at an interval of 4 weeks with 0·5 ml. $^{51}$Cr-labelled red cells from tumour-bearing litter-mate donors 10 days after implantation of latter with tumour R 2426.

A Response to first transfusion.
B Response to second transfusion.

▨ Range of $^{51}$Cr-concentration in normal " August " rats transfused with $^{51}$Cr-labelled cells from litter-mate donors.

tion of $^{51}$Cr on the liver and spleen during the episode. Such agglutinins might be evoked in response to the tumour graft in a manner similar to that in which antibodies to tumour or skin homografts which may be titrated as haemagglutinins are produced in mice (Hildeman and Medawar, 1959). Alternatively, they might be produced by the tumour itself against the host animal's cells as suggested by Green, Wakefield and Littlerwood (1958). In either of these situations, the induction of haemolysis in a normal animal receiving a transfusion of blood from a tumour-bearing donor would be due to the transfer of antibody from the donor's red cells to those of the recipient. However, it has been demonstrated that a haemolytic episode can follow the transfusion of as little as 0·005 ml. of blood and

FIG. 12B.

it appears improbable that sufficient antibody could be transferred by such a mechanism to cause the haemolysis of nearly all the host's cells.

A further possibility is that the tumour might produce an antigen similar or complementary to antigenic components on the host's red cells. Antibodies produced by the host against this antigen might then induce agglutination of the red cells. The antigen could be transferred with the blood of a tumour-bearing donor to a normal recipient and induce a similar production of antibody and agglutination of the recipient's red cells. However, the finding that the time between transfusion and onset of the haemolytic episode may be as little as two days does not seem to be compatible with such a mechanism.

The observations that the haemolytic activity of the blood increases steadily with time after implantation of tumour and that a haemolytic episode can follow the transfusion of minute amounts of blood from a tumour-bearing donor to a normal recipient suggest that the responsible agent is some factor, possibly viral in nature, which is capable of multiplication within the host. If such is the case, the question must be asked whether the virus is the causative agent of the tumour or whether it exists concomitantly in the tumour tissue but without aetiological relationship to it (Zilber, 1958). Further studies designed to test all of these possibilities are being carried out.

### SUMMARY

Rats of the " August " strain bearing the transplantable adenocarcinoma R 2426 undergo an acute haemolytic episode 10–15 days after implantation of tumour. This phenomenon has been investigated by radioactive tracer techniques with $^{51}Cr$ and $^{59}Fe$. Cells are destroyed randomly without regard to age during the haemolytic episode. Destroyed red cells accumulate primarily in the liver and spleen, destruction in the vascular bed of the tumour being absent or extremely small.

A similar haemolytic episode may be induced in normal animals transfused intravenously or intraperitoneally, with blood from tumour-bearing litter-mate donors. The rapidity of onset of the episode is related to the amount of blood transfused and to the time after implantation at which the blood is taken from the donor.

After an initial haemolytic episode induced by implant or transfusion, it is impossible to induce a second episode in the same animal ; furthermore, red cells from a tumour-bearing donor which has suffered a haemolytic episode show near-normal survival when transfused to normal recipients, although they provoke a haemolytic episode in the recipient.

The mechanisms by which these phenomena are brought about are discussed.

This work has been carried out in part at the Institute of Cancer Research, Royal Cancer Hospital, and in part at the Postgraduate Medical School, and has latterly been supported by a grant from the British Empire Cancer Campaign. The technical assistance of Mr. H. C. Christie, Miss Frances Holmes and Miss Judith Topper is acknowledged.

### REFERENCES

BELCHER, E. H.—(1958) 3rd International Symposium on Radioactive Isotopes in Clinical Medicine and Research, Bad Gastein, Munich (Urban and Schwarzenberg), p. 206.—(1959) *Acta Un. int. Cancr*, **15**, 866.

*Idem* AND HARRISS, E. B.—(1957) *J. Physiol.*, **139**, 14.—(1959) *Ibid.*, **146**, 219.

*Idem*, LAMERTON, L. F. AND HARRISS, E. B.—(1958) *Brit. J. Haemat.*, **4**, 390.

BERLIN, N. I., LAWRENCE, J. H. AND LEE, H. C.—(1954) *J. Lab. clin. Med.*, **44**, 860.

BERLIN, R.—(1951) *Acta med. scand.*, **139**, Suppl. 252, p. 1.

DACIES, J. V.—(1954) ' The Haemolytic Anaemias, Congenital and Acquired '. London (Churchill).

EHRENSTEIN, G.—(1958) *Acta physiol. scand.*, **44**, 80.
GREEN, H. N., WAKEFIELD, JUNE AND LITTLERWOOD, G.—(1957) *Brit. med. J.*, ii, 779.
GREENFIELD, R. E., GODFREY, J. E. AND PRICE, V. E.—(1958) *J. nat. Cancer Inst.*, **21**, 641.
*Idem*, STERLING, W. R. AND PRICE, V. E.—(1958) *Ibid.*, **21**, 1099.
HILDEMAN, W. H. AND MEDAWAR, P. B.—(1959) *Immunology*, **2**, 44.
HYMAN, G. A., GELLHORN, A. AND HARVEY, J. L.—(1956) *Blood*, **9**, 618.
JANDL, J. H., JONES, A. R. AND CASTLE, W. B.—(1957) *J. clin. Invest.*, **36**, 1428.
MILLER, A., CHODOS, R. B., EMERSON, C. P. AND ROSS, J. F.—(1956) *Ibid.*, **35**, 1248.
PRICE, V. E. AND GREENFIELD, R. E.—(1958) *Advanc. Cancer Res.*, **5**, 199.
*Iidem*, STERLING, W. R. AND MACCARDLE, R. C.—(1959) *J. nat. Cancer Inst.*, **22**, 877.
ULTMANN, J. E., FISH, W. AND HYMAN, G. A.—(1956) *Cancer Res.*, **16**, 885.
ZILBER, L. A.—(1956) *Advanc. Cancer Res.*, **5**, 291.